# Surface-Based Morphometry of Cortical Thickness and Surface Area Associated with Heschl's Gyri Duplications in 430 Healthy Volunteers

**DOI:** 10.3389/fnhum.2016.00069

**Published:** 2016-03-07

**Authors:** Damien Marie, Sophie Maingault, Fabrice Crivello, Bernard Mazoyer, Nathalie Tzourio-Mazoyer

**Affiliations:** ^1^Groupe d'Imagerie Neurofonctionnelle, Institut des Maladies Neurodégénératives UMR 5293, Université de BordeauxBordeaux, France; ^2^Centre National de la Recherche Scientifique, Groupe d'Imagerie Neurofonctionnelle, Institut des Maladies Neurodégénératives UMR 5293Bordeaux, France; ^3^Commissariat à l'Énergie Atomique et aux Énergies Alternatives, Groupe d'Imagerie Neurofonctionnelle, Institut des Maladies Neurodégénératives UMR 5293Bordeaux, France

**Keywords:** Heschl's gyrus, gyrification, anatomy, MRI, FreeSurfer, cortical thickness, cortical surface area, hemispheric specialization

## Abstract

We applied Surface-Based Morphometry to assess the variations in cortical thickness (CT) and cortical surface area (CSA) in relation to the occurrence of Heschl's gyrus (HG) duplications in each hemisphere. 430 healthy brains that had previously been classified as having a single HG, Common Stem Duplication (CSD) or Complete Posterior Duplication (CPD) in each hemisphere were analyzed. To optimally align the HG area across the different groups of gyrification, we computed a specific surface-based template composed of 40 individuals with a symmetrical HG gyrification pattern (20 single HG, 10 CPD, 10 CSD). After normalizing the 430 participants' T1 images to this specific template, we separately compared the groups constituted of participants with a single HG, CPD, and CSD in each hemisphere. The occurrence of a duplication in either hemisphere was associated with an increase in CT posterior to the primary auditory cortex. This may be the neural support of expertise or great abilities in either speech or music processing domains that were related with duplications by previous studies. A decrease in CSA in the *planum temporale* was detected in cases with duplication in the left hemisphere. In the right hemisphere, a medial decrease in CSA and a lateral increase in CSA were present in HG when a CPD occurred together with an increase in CSA in the depth of the superior temporal sulcus (STS) in CSD compared to a single HG. These variations associated with duplication might be related to the functions that they process jointly within each hemisphere: temporal and speech processing in the left and spectral and music processing in the right.

## Introduction

Heschl's gyrus (HG), which hosts the Primary Auditory Cortex (PAC), exhibits very high anatomical variability in its size and gyrification across both individuals and hemispheres (Penhune et al., 1996; Leonard et al., 1998; Abdul-Kareem and Sluming, 2008; Marie et al., 2015). Three configurations of HG can be observed: a single HG, a Common Stem Duplication (CSD), corresponding to a partial split of the lateral part of the gyrus by the *sulcus intermedius* of Beck, and a Complete Posterior Duplication (CPD) that is a double HG (Abdul-Kareem and Sluming, 2008). Although, the PAC and HG do not exactly overlap at the microscopic level (Morosan et al., 2001). The PAC may seat in the medial two-thirds of a single HG and is limited to the first or anterior HG (aHG) in the case of duplication. MRI methods that provide estimates of myelination have demonstrated a highly myelinated area running over the medial two-thirds of HG in single gyrus and over the aHG in duplications (Dick et al., 2012). This myelinated area likely corresponds to the location of the PAC, primary areas being more heavily myelinated than the associative areas (Glasser and Van Essen, 2011). Using myelin-stained sections, Pfeifer reported one of the first demonstrations of a coupling between acoustic radiations and the gyrus in single HG, while only in aHG in case of duplication (Pfeifer, 1920). Post-mortem probabilistic maps of acoustic radiations further confirmed Pfeifer's pioneering observations (Rademacher et al., 2002).

There are numerous reports of associations between the anatomical characteristics of HG and long-term auditory learning for music and speech, or expertise in either domain (for reviews see Zatorre et al., 2012; Zatorre, 2013). These reports are within the frame of the spectro-temporal framework of acoustic processing (Zatorre and Belin, 2001; Hickok and Poeppel, 2007). For example, Warrier has demonstrated a direct anatomo-functional relationship in HG during sound processing, where the volume of activation during the temporal processing of sound is positively correlated with the volume of aHG on the left but not the right, and the opposite is observed during the spectral processing of sound (Warrier et al., 2009). Schneider combined MRI with MEG to demonstrate a left hemispheric specialization for (missing) fundamental pitch perception and a right hemispheric specialization for spectral pitch perception, consistent with this spectro-temporal framework of acoustic processing (Schneider et al., 2005). In fact, he demonstrated that listeners who completed the pitch change judgments on the fundamental pitch had larger gray matter (GM) volumes in the left HG than did listeners performing the pitch change judgment on the basis of the spectral envelope, who instead had an increase in the right HG GM volume.

Comparisons of the anatomical characteristics of HG with expertise also demonstrated lateralized results (summary in Table 1). In the left hemisphere, a larger volume of white matter (WM) and/or GM of aHG or HG have been associated with good performances in phonological awareness and literacy abilities in 10-year-old children (Sutherland et al., 2012), with expertise or proficiency in phonological processing of foreign speech sounds in adults (Golestani et al., 2007, 2011; Wong et al., 2008) and with early bilingualism (Ressel et al., 2012). Left HG duplications have been associated with phonological expertise (Golestani et al., 2011) but also with phonological disorders and dyslexia (Leonard et al., 1993; Altarelli et al., 2014). Moreover, a large second HG has been reported in dyslexic adults (Leonard et al., 2001).

**Table 1:** **Meta-analysis of reports on HG structure changes related to phonology, music, sound processing, or pathology**.

	**Population**	**Left HG**	**Sound temporal or phonological processing**	**Right HG**	**Sound tonal or musical processing**
Golestani et al., 2011	Adults	↑ Duplication	Expertise phonetics	↑ GM-WM totHG	
		↑ GM–WM totHG			
Sutherland et al., 2012	Children	↑ GM aHG	Phonological awareness, literacy abilities		
Golestani et al., 2007	Adults	↑ WM aHG	Foreign sound learning		
		↑ Duplication			
Wong et al., 2008	Adults	↑ GM-WM aHG	Foreign sound learning		
Ressel et al., 2012	Adults	↑ GM aHG	Early bilinguism		
Schneider et al., 2005	Adults	↑ GM HG-PP	Pitch judgment on fundamental pitch	↑ Duplication	Pitch judgment on spectral envelope
				↑ GM HG-PP	
Leonard et al., 1993	Adults	↑ Duplication	Dyslexia	↑ Duplication	
Leonard et al., 2001	Adults	↑ CSA postHG	Dyslexia		
Ma et al., 2015	Children		Dyslexia	↑ CT	
Altarelli et al., 2014	Boys		Dyslexia	↑ Duplication	
Warrier et al., 2009	Adults	↑ GM–WM aHG	Temporal processing	↑ GM–WM aHG	Spectral processing
				↑ GM medial aHG	AMMA tonal test, N19m–P30m auditory ERP
Bermudez et al., 2009	Adults			↑ GM density	Musical expertise
				↑ CT aHG	
Wengenroth et al., 2013	Adults			↑ GM HG	Musical expertise, AP
Hyde et al., 2009	Children			↑ GM–WM HG^*^	Instrumental training, melodic and rhythmic discrimination test
Foster and Zatorre, 2010	Adults			↑ GM and CT HS	Musical expertise
Liem et al., 2012	Adults			↓ CT HG and HS	N1 auditory ERP

aHG, anterior Heschl's gyrus; AP, Absolute Pitch; CT, Cortical Thickness; ERP, Event-Related Potentials; GM, Gray Matter; HG, Heschl's gyrus; HS, Heschl's sulcus; postHG, posterior Heschl's gyrus in case of duplication; PP, Planum Polare; STG, Superior Temporal Gyrus; totHG, total Heschl's gyrus including both convolutions in case of duplication; WM, White Matter; GM-WM, both GM and WM;

* , the volume was measured using a measure of relative voxel expansion or contraction.

On the right, a larger GM and/or WM volume of HG has been reported in professional musicians with absolute pitch compared to musicians without absolute pitch, and the proficiency of an individual's absolute pitch ability has been shown to be positively correlated with GM volume (Wengenroth et al., 2013). A greater GM density was observed in HG of musicians compared to non-musicians (Bermudez et al., 2009), and a lateral expansion of HG was detected in 6-year-old children who completed 15 months of instrumental training compared to control children (Hyde et al., 2009). Applying surface-based morphometry (SBM) on anatomical images allowed several investigators to report variations in Cortical Thickness (CT) in relation to music expertise or learning in HG (notably reviewed in Zatorre, 2013), such as a CT increase in the right HG associated with musicianship (Bermudez et al., 2009) and pitch discrimination performances (Foster and Zatorre, 2010). Although, less direct, Liem who indicated that the N1 amplitude is an ERP component related to musical expertise, found a negative correlation between N1 amplitude and CT in HG (Liem et al., 2012). Finally, a high occurrence of right duplications had been notably observed in musicians having absolute pitch (Schneider et al., 2005).

The works cited above, which investigated the anatomo-functional relationships in HG in relation to performances during speech or musical sound processing, reported differences in the GM volume, CT, or the occurrence of duplications. Although we have recently shown that the presence of duplications is associated with a decrease in the surface area of aHG (Marie et al., 2015), the changes in CT or Cortical Surface Area (CSA) associated with the occurrence of duplications are still unknown. We applied SBM to the anatomical images of 430 participants who had previously been characterized according to their HG macroscopical anatomy (single HG, CSD, and CPD, Marie et al., 2015) to characterize the differences in CT and CSA in groups with varying HG duplication patterns.

## Materials and methods

### Participants

The characteristics of the 430 participants are fully described in our previous article (Marie et al., 2015). All were free of brain abnormalities, and the Basse-Normandie's ethical committee approved the study. All participants provided their informed written consent and received compensation for their participation in the framework of the BIL&GIN database (Mazoyer et al., 2015). Briefly, the population is balanced for handedness (self-report, 232 right-handers vs. 198 left-handers, Chi square = 2.69, *p* = 0.10), and gender (209 men vs. 221 women, Chi square = 0.33, *p* = 0.56). The mean age of the participants is 26 years (*SD*, 8). Each volunteer's skull perimeter was measured at the level of the eyebrows, passing at the top edge of the ears and along the occipital bump. The mean skull perimeter was 57 cm (*SD*, 2) and men had larger skull perimeters than women did (+3 cm, *t* = 17.1, *p* < 0.0001). Note, that none of these individual variables differed according to the left or right HG number (Marie et al., 2015).

### Image acquisition

Anatomical images were acquired from 2007 to 2011 using the same 3T Philips Achieva scanner (Philips Medical Systems, Best, The Netherlands). High-resolution T1-weighted images were obtained using a 3D-FFE-TFE sequence (TR, 20 ms; TE, 4.6 ms; flip angle, 10°, inversion time, 800 ms; turbo field echo factor, 65; Sense factor, 2; field of view, 256 × 256 × 180 mm; and isotropic voxel, 1 mm^3^). For each participant, the line between the anterior and posterior commissures was identified on the midsagittal plane, and the T1-MRI volume was acquired after orienting the brain in the bi-commissural coordinate system.

### Image analysis

#### Definition of the HG gyrification patterns

A single expert (NT-M) identified HG and its gyrification type on individual images in the native space as fully described elsewhere (Marie et al., 2015). Briefly, the definition of the number of HG was optimized via the knife-cut method, which is based on the reconstruction of an oblique section plane passing through the Sylvian fissure (Kulynych et al., 1993; Tzourio-Mazoyer et al., 2010) computed with the aid of dedicated software (Voxeline, Diallo et al., 1998). An oblique section plane parallel to the Sylvian fissure was generated by uncovering the superior temporal plane and passing through the point where HG is the largest. Axial, coronal, sagittal, and oblique slices are displayed simultaneously for identifying duplications.

The gyrification pattern of HG (single HG, common stem duplication or CSD, or complete posterior duplication, or CPD) was defined according to the anatomical criteria reviewed in Abdul-Kareem and Sluming (2008). In cases with a single HG, the anterior border is constituted by the temporal transverse sulcus, and the posterior border is Heschl's sulcus. In cases with a CSD, the gyrus is partially split by the *sulcus intermedius* of Beck (SI), which never reaches the internal border of the gyrus. We considered a CSD to be present if the SI length was at least one-third of the length of HG. There are two Heschl's sulci in cases with a CPD; the first Heschl's sulcus splits the gyrus medially into two parts and the second Heschl's sulcus becomes the posterior border of HG. As previously reported, we identified 284 single HG (66%), 98 CSDs (23%), and 48 CPDs (11%) in the left hemisphere, and 239 single HG (56%), 95 CSDs (22%), and 96 CPDs (22%) in the right hemisphere (Marie et al., 2015).

#### Reproducibility of the identification of the gyrification pattern

Reproducibility was measured by a second assessment of 40 randomly selected brains (80 hemispheres) by the same observer. The concordance between the two evaluations was measured with the chance corrected Kappa statistics (JMP pro 11). The Kappa statistics for the concordance of the two assessments of the number of gyri was 0.84 ± 0.06 (*p* < 0.0001). The Kappa statistics for the concordance of the two assessments of the HG pattern (single, CPD or CSD) was 0.82 ± 0.06 (*p* < 0.0001). According to Cohen's original paper, a Kappa value > 0.81 attests an excellent reproducibility (Cohen, 1960). Non-concordant classification occurred in eight cases (10% of the hemispheres). Four of the non-concordant associations corresponded to a confusion between CPD and CSD; in two cases, the non-concordance was between CSD and single, and in two others, it was between single and CPD in the right hemisphere.

#### Surface-based morphometry

The cortical surfaces were reconstructed and the anatomical features were measured for each participant with the FreeSurfer 5.3.0 image analysis suite (http://surfer.nmr.mgh.harvard.edu/, (Dale et al., 1999; Fischl et al., 1999a,b, 2001, 2002, 2004; Fischl and Dale, 2000; Fischl, 2012). The FreeSurfer analysis stream includes intensity bias field removal optimized for the present 3T acquisitions and skull stripping. In the native anatomical space of the subject, FreeSurfer constructs models of the cortical surface that can be represented as a mesh of triangles and tessellated into over 160,000 vertices for each hemisphere. The vertex positions are adjusted such that the surface follows the T1 intensity gradient between the cortical WM and GM. This highly folded surface, called “white surface,” can be inflated to see buried surfaces within the sulci. A second surface, called the “pial surface,” is fit to the outer GM surface of the brain. The hemispheres are processed separately through a precise estimation of the inter-hemispheric fissure plane.

#### Quality control

**Figure 1:**
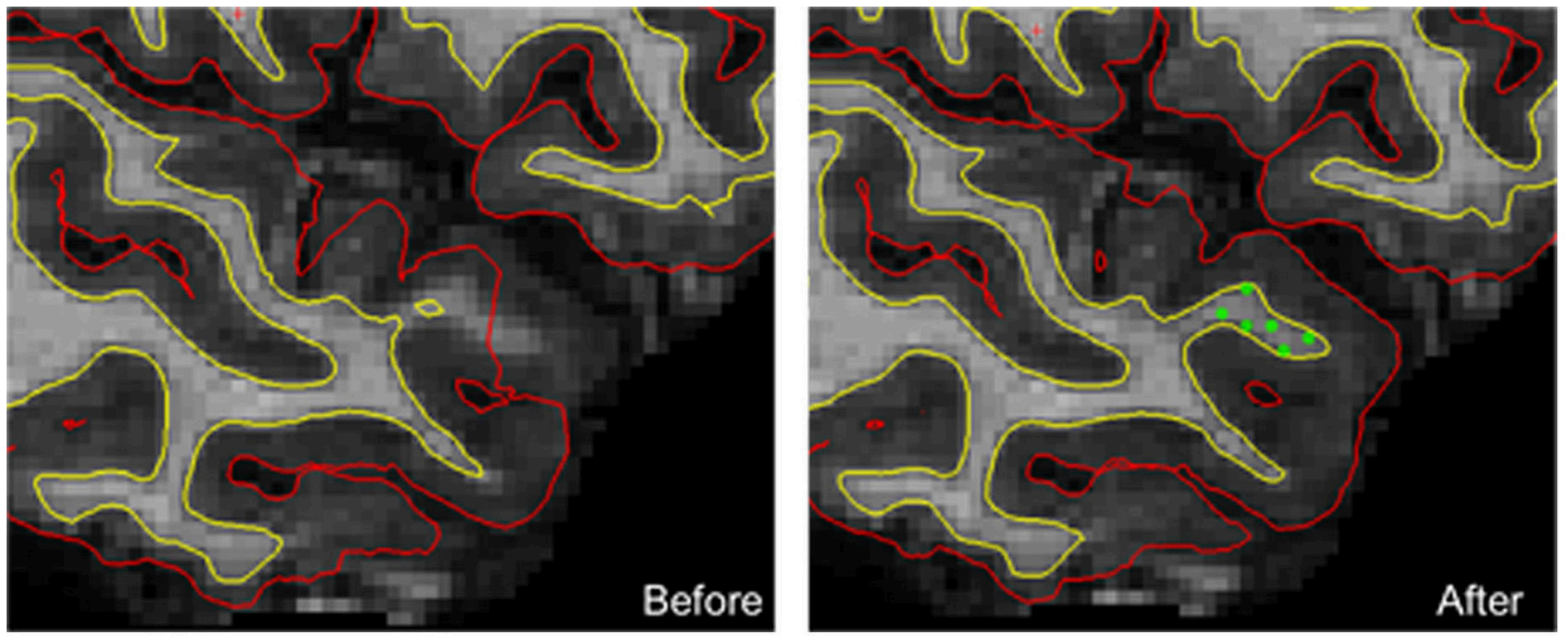
**An example of manual corrections applied in case of wrong estimation of pial and/or white cortical surfaces**. In this individual, the FreeSurfer software did not correctly estimate both white and pial surfaces in the upper part of the temporal pole. Manually adding checkpoints to the white matter surface results in better surface estimation after a second extraction process (yellow line, white surface; red line, pial surface; green dots, checkpoints).

The problems of estimating the surfaces around the thin temporal lobe strands that border the tip of the lateral ventricle have been identified with the FreeSurfer segmentation procedure (Dale et al., 1999). In this case, a reduction of the intensity of the voxels in the corresponding white matter leads to a misclassification of voxels and an inaccurate definition of the white and pial surfaces. To overcome this limitation, we visually checked (SM) each of the 430 pial and white surfaces on each axial, sagittal, and coronal section from each participant twice and identified surface reconstruction errors in 44 cases (10%). A correction procedure consisting of manually adding an average of nine control points (*SD*, 7) in the misclassified white matter was then applied in the 44 individuals. Then, the FreeSurfer procedure was launched a second time for these corrected individuals, resulting in more accurate surface reconstructions (Figure 1).

#### Calculation of the morphometric variables in the native space of each participant

For each vertex, CT is defined as the distance between the white and pial surfaces and CSA is defined as the average area of the triangles to which the vertex belongs. The cortical surface curvature (CURV) parameter was also extracted and used to drive the non-linear surface-based inter-subject registration. The curvature is estimated as 1/*r*, where *r* is the radius of the inscribed circle to each point (or vertex) of the white surface.

#### Definition of a specific template for between-subject registration and smoothing

A specific surface-based template for between-subject registration that included images from individuals with identical HG duplication patterns in the left and right hemispheres was computed to avoid a left alignment weighted toward the single HG scenario and a right alignment weighted toward the duplication scenario (HG anatomical pattern in Fsaverage, see Supplementary Figure 1). Forty individuals of the sample were included (26 men, 18 right-handers), corresponding to 20 bilateral single HG and 20 bilateral duplications (10 CSD and 10 CPD). The procedure for computing this specific surface-based template, called 40-average, is fully described and illustrated in Supplementary Material and Supplementary Figure 2.

Non-linear surface-based normalization of the 430 CT and CSA individual maps to this specific template was then applied. An additional step was performed by adding a Jacobian correction to the CSA maps to account for any stretching or compression in the registration that occurred during the nonlinear registration (Winkler et al., 2012). Note, that this correction is not needed for the CT maps because CT is measured along a vector normal to any stretching or compression.

After registration, the individual CT and CSA maps were surface-smoothed by a 10 mm FWHM Gaussian filter size, statistical power of both features benefiting from such optimal smoothing filter size as demonstrated by Liem et al. (2015). However, minimal detectable differences depending on smoothing filter size (Liem et al., 2015), individual CT maps of two groups (left CPD, and left single HG) were also surface-smoothed with a 5 mm FWHM Gaussian filter in a complementary analysis in order to evaluate the impact of a reduced filter size on structural differences detected between HG anatomical patterns.

#### Curvature variance maps

To characterize the intrinsic anatomical variability of each HG configuration, we computed CURV variance maps in the 40-average space in both hemispheres. For each vertex, we calculated a CURV variance value using MATLAB 7.11.1 (R2010b), which was defined as the mean of the square minus the square of the mean for a given group (for example the calculation of curvature variance in the left hemisphere for the 284 subjects with single HG).

### Statistical analysis

To compare the groups with varying HG duplication patterns, we ran two independent ANCOVA in each hemisphere on CT and CSA separately, with the HG pattern as the main factor. All analyses were performed with the QDEC module included in FreeSurfer, and the participant's skull perimeter was included as a confounding variable to account for differences in head size. The statistical threshold was set at *p* < 0.05 FDR corrected, and an additional cluster extension threshold was set at 20 mm^2^.

To display the results on the supratemporal plane, a specific patch of the temporal lobe was computed for the mean image of each group and hemisphere by merging the corresponding labels of Desikan's parcellation provided by FreeSurfer (Desikan et al., 2006). The removal of the fusiform, entorhinal, and parahippocampal gyri was performed to clean up the patch (**Figure 3**).

Finally, the overlap of the significant variations in CT and CSA were computed by summing the binarized image of each anatomical feature (**Figure 4**).

## Results

### Macroscopic anatomical variability of HG configuration across hemispheres: analysis of the CURV variance maps

**Figure 2:**
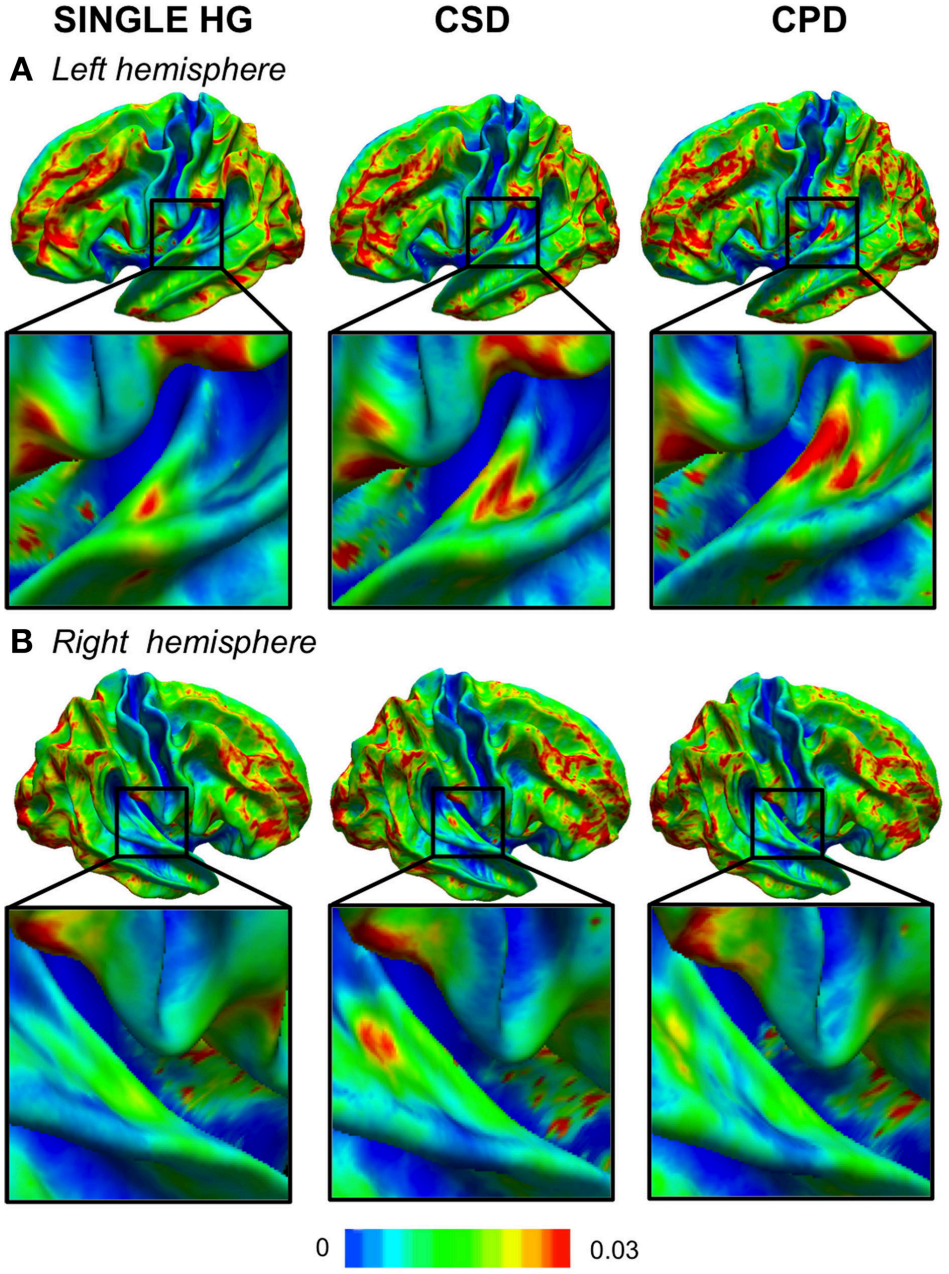
**Variability maps of the curvature (CURV) parameter in the left (A) and right (B) hemispheres**. The color scale represents the variance of the CURV parameters computed for each HG gyrification pattern in each hemisphere. The zoomed part is centered in the temporal transverse region where the HG duplication is located.

In cases with a single HG, the variability was low, except in the antero-lateral border of the left HG (Figure 2A). In cases with a CSD in the right hemisphere, a small cluster of high variability was observed in the *sulcus intermedius* of Beck (Figure 2B), similar to that on the left, which corresponded to a variation in the width of the mid-part of the second sulcus. A high variability of left CPD was observed and corresponded to the variation in the curvature pattern of the posterior position of the first Heschl's sulcus. On the right, a comparable pattern was present, but with a much lower amplitude.

### Morphometric changes according to the duplication types

#### CT differences

**Figure 3:**
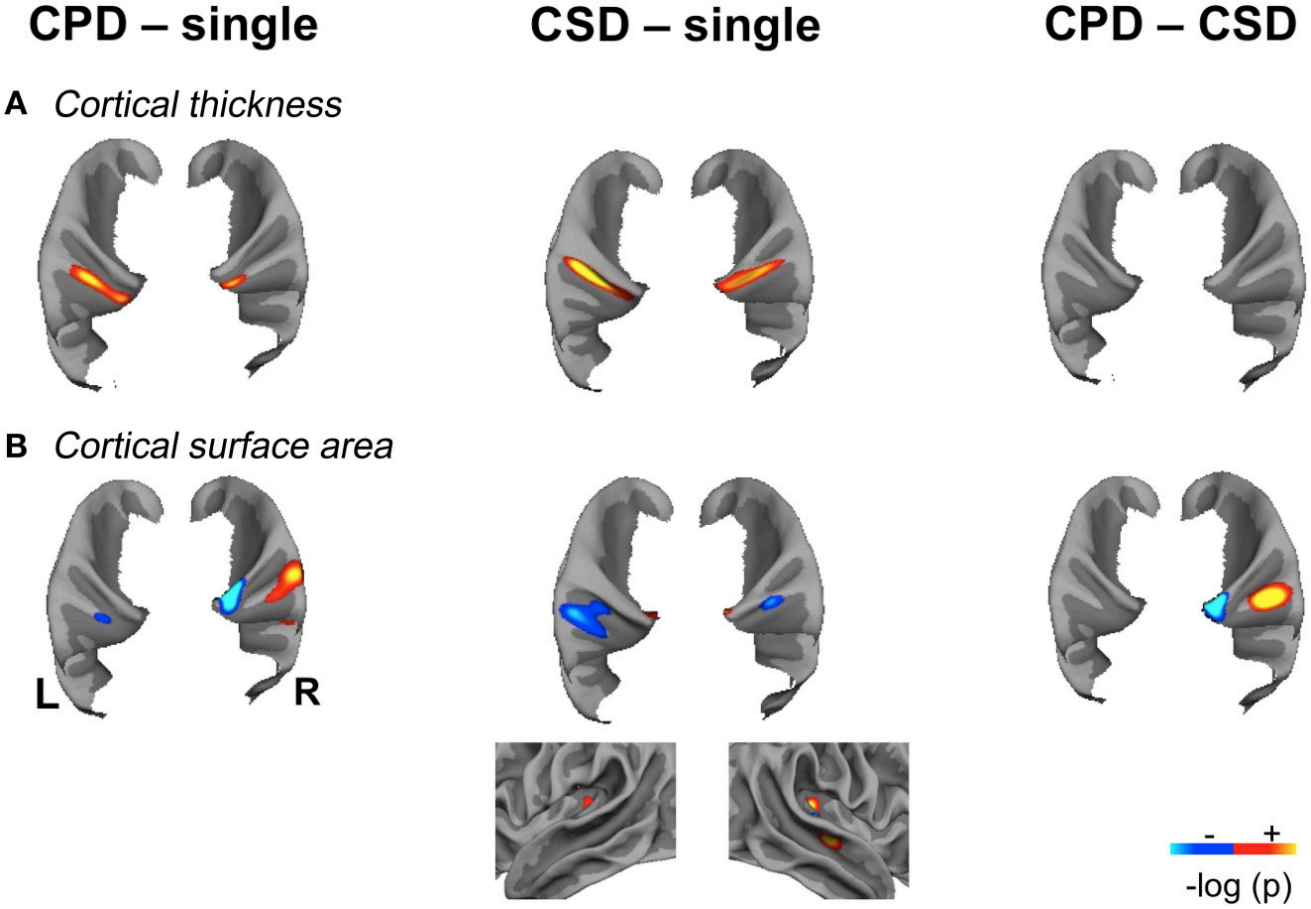
**Significant variations of Cortical Thickness (CT) and Cortical Surface Area (CSA) in the “CPD–single HG”, “CSD–single HG” and “CPD–CSD” contrasts in the left and right hemispheres showing the spatial overlap of CT and CSA variations. (A)**. Significant variations in cortical thickness. **(B)**. Significant variations in cortical surface area, the lateral view of the right temporal lobe is provided to illustrate the increase in CSA located in the mid-part of the STS in the “CSD–single HG” comparison. Significant variations are superimposed on the mean patch of each corresponding group (“CPD–single” and “CPD–CSD” contrasts: mean patch of 48 left CPD, mean patch of 96 right CPD; “CSD–single HG” contrast: mean patch of 98 left CSD and 95 right CSD; CSA: cortical surface area; CT, cortical thickness; CPD, common posterior duplication; CSD, common stem duplication; HG, Heschl's gyrus; *p* < 0.05 FDR corrected for multiple comparison; cluster extension threshold, 20 mm^2^; hot color indicates a positive variation; cold color, indicates a negative variation; light gray, gyri; dark gray, sulci).

A larger CT was observed in the CSD group compared to the single HG group all along Heschl's sulcus and the second gyrus (Table 2, Figure 3).

**Table 2:** **Local variations of Cortical Surface Area (CSA) detected in “CPD–single HG”, “CSD–single HG,” and “CPD–CSD” contrasts**.

**Anatomical localization**	**Cluster size (mm^2^)**	***p*_*max*_**	***x***	***y***	***z***	**Dupl. mean (*SD*) (mm^2^)**	**single HG mean (*SD*) (mm^2^)**
**L: CPD–SINGLE HG**							
Medial 2^nd^ HG	27	3.10^−6^	−46	−30	7	26 (4)	30 (5)
**R: CPD–SINGLE HG**							
Medial aHG	161	9.10^−9^	40	−23	10	155 (28)	176 (31)
Lateral 2^nd^ HG	176	10^−6^	64	−18	6	192 (31)	171 (27)
**L: CSD–SINGLE HG**							
HS-PT	187	2.10^−6^	−58	−28	8	197 (33)	214 (36)
Medial TTS	52	3.10^−5^	−30	−27	12	60 (7)	57 (6)
**R: CSD–SINGLE HG**							
Middle STS	172	3.10^−7^	53	−17	−3	193 (29)	177 (28)
Medial TTS	88	8.10^−7^	32	−26	14	95 (8)	91 (8)
HS	43	3.10^−6^	49	−24	7	39 (8)	45 (8)
**R: CPD–CSD**							
Medial aHG	126	4.10^−10^	38	−25	13	126 (19)	145 (23)
Lateral 2^nd^ HG	156	4.10^−8^	57	−25	10	188 (34)	159 (26)

Dupl., duplication; HG, Heschl's gyrus; aHG, anterior Heschl's gyrus; HS, Heschl's sulcus; PT, planum temporale; TTS, temporal transverse sulcus; SD, Standard Deviation; STS, superior temporal sulcus; p < 0.05 FDR corrected for multiple comparison; cluster extension threshold, 20 mm^2^; x, y, z, Talairach coordinates rounded to the whole number.

A larger CT was also observed in the medial part of the second HG in both hemispheres in the CPD group compared to the single HG group, but it was more medially situated on the right.

Note, that there was no difference between CSD and CPD.

**Figure 4:**
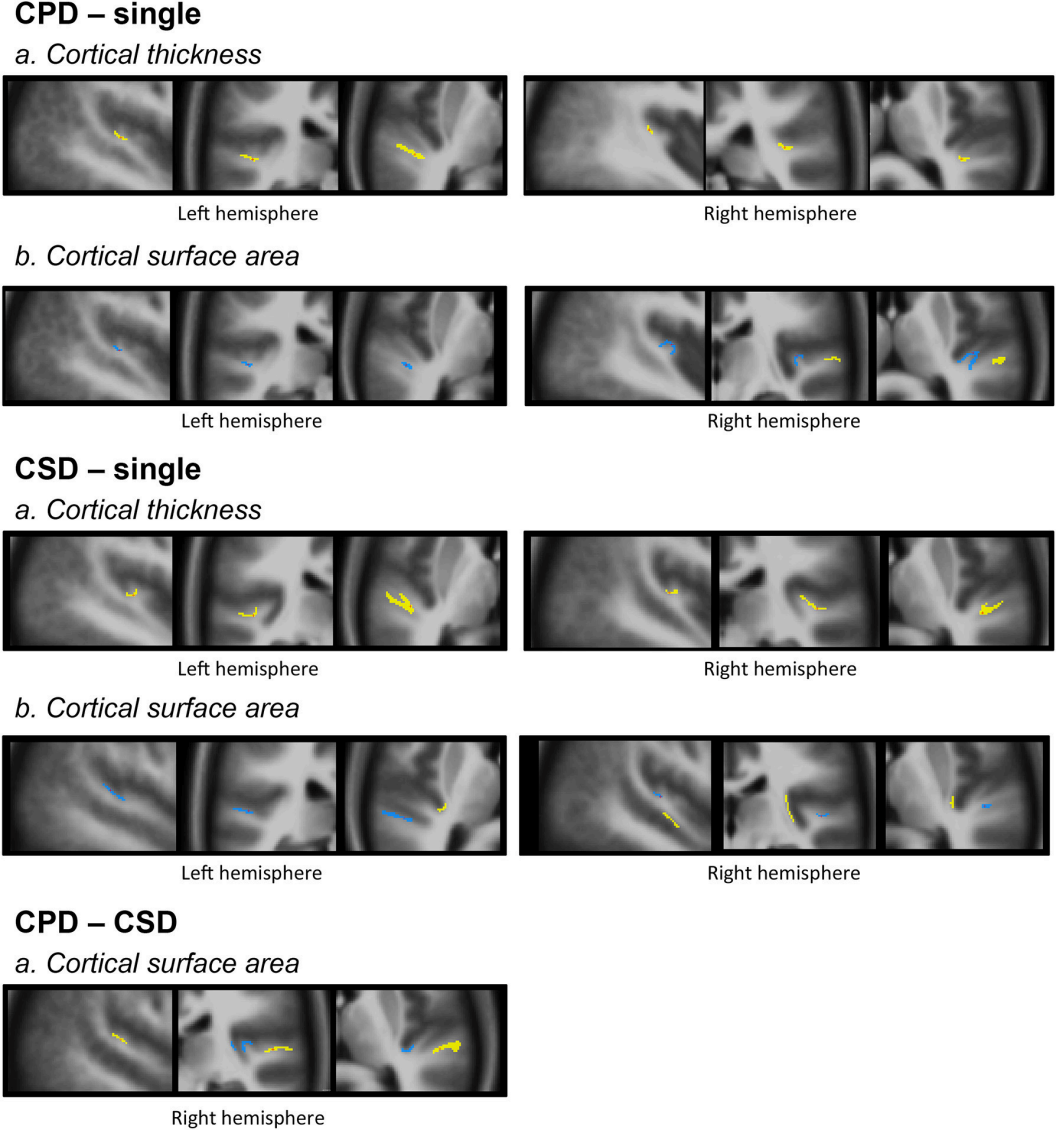
**Morphology and relationships between variations in CT and CSA**. Projection of the SBM results on the volumetric means of each group of individuals defined according to their gyrification pattern. Results of “CPD-single” and “CPD-CSD” contrasts superposed on the mean images of 48 left CPD or 96 CPD for the right side. Results of “CSD-single HG” contrasts superimposed on the mean image of 98 CSD for the left hemisphere or 95 CSD for the right hemisphere. The yellow scale corresponds to increased values, and the blue scale to decreased values. From left to right a sagittal, a coronal, and an axial slice are presented passing through the significant clusters.

These increases in CT in case of duplication, whatever the side and whatever the type of comparison (either CSD or CPD minus single), were located at the ventral limit of the second gyrus. In addition, in case of CSD, the increase in CT extended from the ventral part of the second gyrus toward the *sulcus intermedius* of Beck (Figure 4).

#### CSA differences

In the right hemisphere, the CSA was significantly smaller in the medial part of aHG in the CPD group compared with the single HG or CSD groups, whereas a larger CSA was observed in the lateral part of the second HG of the CPD group compared with the other two groups (Table 3, Figure 3). In the left hemisphere, a decreased CSA was detected on the medial part of the second gyrus of CPD as compared to the single HG group, corresponding to the location of the planum temporale (PT) in single HG cases.

**Table 3:** **Local variations of Cortical Thickness (CT) detected in “CPD–single HG”, “CSD–single HG” and “CPD–CSD” contrasts**.

**Anatomical localization**	**Cluster size (mm^2^)**	***p*_*max*_**	***x***	***y***	***z***	**Dupl. mean (*SD*) (mm)**	**Single HG mean (*SD*) (mm)**
**L: CPD–SINGLE HG**							
2^nd^ HG	143	6.10^−7^	−48	−28	6	2.70 (0.26)	2.43 (0.25)
**R: CPD–SINGLE HG**							
2^nd^ HG	36	3.10^−7^	42	−31	12	2.71 (0.34)	2.44 (0.32)
**L: CSD–SINGLE HG**							
HS	208	3.10^−9^	−49	−24	5	2.72 (0.26)	2.52 (0.25)
**R: CSD–SINGLE HG**							
Medial 2^nd^ HG–HS	145	6.10^−7^	46	−25	8	2.80 (0.24)	2.62 (0.26)

Dupl., duplication; HG, Heschl's gyrus; HS, Heschl's sulcus; SD, Standard Deviation; p < 0.05 FDR corrected for multiple comparison; cluster extension threshold, 20 mm^2^; x, y, z, Talairach coordinates rounded to the whole number.

In the middle part of Heschl's sulcus, smaller CSA values were observed in both hemispheres of the CSD group compared to the single HG group. Interestingly, the extent of the cluster was larger on the left and expanded in the PT.

In the medial part of the temporal transverse sulcus, a larger CSA was also detected in both hemispheres of the CSD group compared to the single HG group. Moreover, in the right hemisphere, a larger CSA was observed directly under the lateral part of HG in the depth of the middle part of the Superior Temporal Sulcus (STS) (Figure 3).

#### Spatial overlap between the CSA and CT variations

The decreased CSA observed in the left and right Heschl's sulci overlapped with the area of the increased CT in both the CPD minus single and CSD minus single comparisons (Figure 4).

## Discussion

Surface-Based Morphometry allowed us to investigate changes in macroscopical neuroanatomy without being limited to the intrinsic voxel resolution (Fischl and Dale, 2000). The high spatial normalization provided by the surface-based inter-subject alignment of curvature in Freesurfer enabled us to describe the changes in CT and CSA associated with the HG gyrification patterns, completing the results previously obtained by measuring the aHG surface area using manual delineation (Marie et al., 2015).

### Methodological considerations

The identification of the HG gyrification patterns had an excellent reproducibility according to the Kappa measure. A first type of non-concordance was related to the distinction between a single gyrus and a CSD because the criterion for CSD was the minimal length of the sulcus intermedius of Beck (30%). A second type of non-concordance was the confusion between CPD and CSD, which was related to the determination of whether the sulcus splitting the gyrus reaches the internal limit of HG. This ambiguity is generally solved by applying the second criteria based on the shape of the duplication, which resembles an “M” on sagittal and coronal slices in cases with a CPD or a “heart” in cases with a CSD. The two cases of confusion between single and CPD were located in the right hemisphere, and corresponded to a difficulty in discriminating a second gyrus from the right PT.

The second methodological point concerns the use of a specific surface-based template in the present study. While we revealed that the use of such specific template improved the inter-subject registration of HG anatomical pattern, in particular for the group of individuals presenting a complete HG duplication in the left hemisphere (see mean images presented in Supplementary Material and Supplementary Figure 1 for a detailed description of this improvement), we also showed, that the use of such specific template did not modulate the statistical differences detected between groups for both CT and CSA anatomical (see Supplementary Material and Supplementary Figure 3). The present absence of differences between results detected by statistical analyses obtained after a normalization performed with the specific 40-average template and the default Fsaverage template strongly suggests that the accurate alignment of HG anatomical pattern obtained with the specific 40-average surface-based template has no influence on the statistical maps resulting from group comparisons, mainly due to the lower spatial resolution of these statistical maps as compared to the resolution of the mean curvatures images. In addition, the results we obtained in the CPD minus single HG analysis with a smoothing filter size of 5 mm are similar to the results deriving from the analysis performed with a 10 mm smoothing filter, suggesting there is no impact of the size of the smoothing filter on CT variations related to HG structure changes (see Supplementary Results).

### Variability of each HG pattern

We conducted an analysis of variance on CURV, which clearly showed a high variance on the left, increasing from single to CSD to CPD. In fact, difference of variability between hemispheres might not be significant (due to the absence of any equivalence between the vertices of the two hemispheres, statistical comparison was not possible). In both hemispheres, a high variance was located in the most lateral part of HG for the single group and in the medial part for the CSD and CPD groups, and a low variance was observed in the internal part of HG. In the stereotactic space, Leonard et al. described the probability of the position of the *sulcus intermedius* of Beck and the second Heschl's sulcus in cases with duplications. They demonstrated differences in the position of the second sulcus across hemispheres, with a second sulcus that does not angle as sharply on the left as it does on the right (Figure 6 of Leonard et al., 1998), but they did not report any difference in the variability of sulci positions across hemispheres.

### Morphological support of CT and CSA variations

The present work report both local differences directly related to morphological changes associated with duplication and more distant variations that may relate to differences in anatomical and/or functional organization associated with differences in gyrification. The location of CT increases, consistently located at the second gyrus in case of CPD, and along the sulcus intermediary of Beck in case of CSD, suggests that it corresponds to a morphological change from Heschl's sulcus to the second (complete or partial) gyrus. As it had been underline by Van Essen (1997), CT might be larger in top of gyri than in the depth of sulci.

Concerning CSA the picture was different depending on hemispheres and on the type of duplication. The modifications we report could not be attributed solely to local morphological changes in relation with differences in gyrification for HG.

### Opposite pattern of modification between CT and CSA differences

Interestingly, when an overlap of CT and CSA modifications occurred, it always corresponded to an increase of CT in case of duplication associated with a local decrease of CSA, an opposite profile of structural modification of these two anatomical features. These results have to be linked to the study of Hogstrom on local correlations of CT and CSA through the whole cortex. By studying 322 healthy adults aged from 20 to 85 years old thanks to surface-based method, they observed that CSA and CT were negatively correlated in a per-vertex general linear model that took into account the age and gender of the participants (Hogstrom et al., 2013), corresponding, as in the present study, to opposite differences in CT and CSA variations.

### Duplications are associated with a larger CT in the second HG: a candidate support for increased abilities associated with duplications in healthy individuals

Bilateral increases in the CT could represent a morphological support for the great abilities reported in individuals having duplications. As reviewed in the introduction, expertise in pitch processing based on the spectral envelope has been associated with a right duplication (Schneider et al., 2005), while the ability to rapidly learn foreign speech sounds (Golestani et al., 2007) and phonetic expertise (Golestani et al., 2011) were related to a left duplication. Musicianship was also associated to a larger cortical thickness all along the posterior supra-temporal plane surface posterior to HG, thereby including the second gyrus of duplication, when one is present (Bermudez et al., 2009). Finally, the CT of the right Heschl's sulcus has been positively associated with performances in a relative pitch detection task (Foster and Zatorre, 2010). The locations across studies are consistent and the larger CT we observed in the present study overlapping with the increased CT reported by Foster's study in cases with a CSD, whereas it was in a slightly posterior position in cases with a CPD (Foster and Zatorre, 2010). According to the literature, increase in CT could relate to an increased number and/or size of cells within a column, their packing density, or the number of connections and extent of myelination (Rakic, 1995; Eickhoff et al., 2005).

On the other hand, the increased CT associated with duplications that we report here is similar to the CT of the Heschl's sulcus label of the Destrieux parcellation (Destrieux et al., 2010), which has been shown to be anti-correlated with the amplitude of the N1 component of auditory-evoked potentials in the right hemisphere (Liem et al., 2012). As reviewed by Liem and colleagues, the N1 amplitude reflects the first step of auditory processing and is modulated by auditory experience, such as musical training (Pantev et al., 2001; Baumann et al., 2008). Liem et al. (2012) postulated that a refinement in the synaptic circuitry might have resulted in a thinner cortex, allowing neurons to be better synchronized and generate the electrophysiological signal. This hypothesis is in accord with Hyde's proposal that a thicker cortex might be less efficient in terms of neural processing (Hyde et al., 2007). Hyde et al. reported a thicker cortex in the right inferior frontal gyrus and the right auditory cortex in amusic patients compared to musically intact controls, and suggested that they were related to cortical malformations in the amusic brain. A recent study in dyslexics also showed an increase in the CT of the right superior temporal gyrus extending to HG and the PT, which resulted in a rightward CT asymmetry that was different from the healthy volunteers (Ma et al., 2015).

Thus, the increased CT observed in cases with HG duplications may or may not represent an advantage, depending on the population studied. A thicker cortex could be associated with cognitive impairments in cases with abnormal development, while, on the other hand, it would be associated with improved abilities in the context of normal development. This hypothesis needs further exploration, but it could partially explain the apparently contradictory associations of HG duplications with both expertise and dyslexia (Leonard et al., 1993, 1995, 1998, 2001; Altarelli et al., 2014).

### The left CSD is associated with a smaller CSA in the PT, while, on the right, it is associated with a larger CSA in the STS

In our previous work using manual delineation of the aHG (Marie et al., 2015), we have shown that left duplication is associated with decreased left aHG surface area. The present study also shows that left duplication is associated with a decreased left PT surface area. Such an association might have been previously overlooked because the second HG is usually included in the PT in cases with a complete duplication (as reviewed by Shapleske et al., 1999). Decrease of left aHG size had been associated with a lower leftward lateralization of the temporal processing sounds (Warrier et al., 2009), and indirectly with lateralization during speech listening (Tzourio-Mazoyer et al., 2015), while a lower left PT surface area had also been associated with lower leftward asymmetry during speech listening (Tzourio et al., 1998; Josse et al., 2003, 2006). The question of whether these decreased surface areas and related anatomical asymmetries are detrimental to speech perception in healthy subjects is an open issue; the work conducted by Leonard and others suggests, that it might be the case in dyslexics that exhibit more HG duplications (Leonard et al., 1993, 1995, 1998, 2001; Altarelli et al., 2014).

Increased CSA was located in the middle part of the STS when a right CSD occurred. STS is rightward asymmetrical for gray matter volume (Watkins et al., 2001; Barrick et al., 2005) and sulcal depth (Van Essen, 2005; Leroy et al., 2015). Human-specific rightward STS asymmetry is organized early in development, as shown by Glasel et al. (2011). The rightward asymmetries of STS described in the abovementioned works are located ventrally to the right HG, as in the present study. From a functional point of view, the right HG, and STS are two major anatomical supports of the spectral processing and pitch analysis, and they constitute the neural support of the analysis of spectral envelope independently of the fine spectro-temporal structure. Warren suggested, that STS may have a generic role in the spectral analysis of sound (Warren et al., 2005) and specified that this role did not exclude the possibility that specific subregions of the STS are functionally specialized for higher order analysis of specific sound classes. In fact, the middle STS has been involved in syllables (Jäncke et al., 2002; Poeppel et al., 2004), speech (Binder et al., 2000), and voice processing (Belin et al., 2000). Interestingly, one study showed that the acoustic parameters of voices are extracted in the right middle STS for vocal tract parameters, and in the right HG for glottal fold parameters (Kreitewolf et al., 2014). Because these two distinct anatomical areas could share a close functional role, one might suggest they have associated anatomical variations. Regarding the diversity of auditory processing mediated by the middle part of the STS, further studies are needed to conclusively determine the functional correlates of this increase in CSA in the middle STS in cases with a CSD.

Compared with a single HG in both hemispheres, CSD was also characterized by an increase of medial CSA at the interface between the temporal transverse sulcus and aHG. These increases in the CSA could relate to the growth of the wide common stem supporting the two merging gyri in cases with a CSD.

### CPD and CSD exhibit anatomical differences in the CSA in the right aHG

The differences across duplication patterns were limited to variations in CSA on the right. This result is only partially consistent with our previous report of an absence of significant differences in the reduced surface area of aHG between the CSD and CPD groups (Marie et al., 2015), that might be in relation to the increased sensitivity provided by the examination of CSA extracted by SBM as compared to manual delineation. aHG and the PAC primarily overlap in the medial part of aHG (Penhune et al., 1996; Rademacher et al., 2001), where a decreased CSA was observed in the CPD group. This decrease may correspond to a decrease in the number/spacing of the minicolumns of the PAC, according to the radial-unit hypothesis of cortical development initially proposed by Rakic (1995, 2009) and acknowledged as a potential interpretation of such variations by others (Elmer et al., 2013; Meyer et al., 2013). The radial-unit hypothesis postulates that the morphology of the CSA of a given brain region is principally driven by the number and spacing of the ontogenetic or radial cortical columns, rather than by the number and size of cells within a column, packing density, or number of connections (Rakic, 1995; Eickhoff et al., 2005), a functional cortical column of the brain corresponding to several radial columns (Rakic, 1988, 2009). Under this hypothesis, CSA variations presently observed could relate to changes in the number and spacing of minicolumns and, consequently, to differences in processing power. Further investigations are needed to test such hypothesis and evaluate the potential behavioral correlates. Actually there is no consensus regarding the underlying cellular mechanisms associated with variations in CSA, although numerous mechanisms such as neurogenesis or myelin changes have been proposed as potential candidates for CT/gray matter, or white matter variations, respectively, (Draganski and May, 2008; Zatorre et al., 2012). This lack of knowledge applies also to gray matter density evaluated by VBM that does not correlate with histological measurements of neuronal density (Eriksson et al., 2009) pointing toward a need for understanding of the microstructural changes that underlie these macrostructural variations detected by computational neuroanatomy.

Laterally, a larger right CSA was observed in the second gyrus of the CPD group compared with both the CSD group and the single HG group. This location corresponds to the PT and the second HG of CPD, which is considered to belong to the PT when present (as reviewed by Shapleske et al., 1999). On the right side, the space devoted to the supratemporal plane, which includes HG and the PT, is smaller than that on the left side (Toga and Thompson, 2003), and CPD are more frequent (Marie et al., 2015). The present results indicate, that the increased surface area of the second HG might actually be at the cost of the right PT surface area when a CPD occurs.

## Conclusions

The present SBM study conducted in 430 participants explored the changes in CT and CSA associated with HG duplications. Variations, which significance and location were independent of the template used and of the smoothing of the images, were observed in HG and surrounding areas, i.e., the left PT and the right mid STS. Despite the lack of consensus on the neurophysiological basis of such morphological features changes measured with structural MRI, the present findings provide important information for neuroimaging research led on inter-individual anatomical variability of HG in relation to speech and/or music processing, their dysfunctions, as well as training and expertise in these domains.

## Author contributions

All authors listed, have made substantial, direct and intellectual contribution to the work, and approved it for publication.

## Funding

This work has been funded by our guardianships: the CNRS, CEA, and University of Bordeaux and DM was supported by a grant from the Aquitaine Regional Council (France).

### Conflict of interest statement

The authors declare that the research was conducted in the absence of any commercial or financial relationships that could be construed as a potential conflict of interest.
