# Supplementary material for: Surface-Based Morphometry of Cortical Thickness and Surface Area Associated with Heschl's Gyri Duplications in 430 Healthy Volunteers
Source: Front Hum Neurosci. 2016 Mar 7;10:69. doi: 10.3389/fnhum.2016.00069 (PMC4779901; doi:10.3389/fnhum.2016.00069)
Supplement: Supplementary file 1 [file SupplementaryMaterial.docx]

**Supplementary material**

**Method**

*Definition of a specific template for between-subject registration*

Because of the unequal distribution of duplications across hemisphere in the general population, it was necessary to ensure that the FreeSurfer default procedure correctly estimated the morphology of HG. To do so, we first used Voxel-Based Morphometry (VBM) to compute mean images of groups with varying duplications. More precisely, 6 mean images (left single HG, left CSD, left CPD and the same for the right hemisphere, Supplementary Figure 1a) obtained with VBM inter-subject registration were computed using SPM5 software. The individual T1-weighted volumes had previously been spatially normalized to the reference template from the BIL&GIN database, as described elsewhere (Mazoyer et al., 2015). A similar approach was then applied using FreeSurfer procedure by computing the same set of 6 mean images that had been surface-based registered in the Fsaverage default surface template. The curvature values were displayed on the mean morphology to have a better view of the gyri (red) and sulci (blue) (Supplementary Figure 1) and visually compare these mean surface-based images to the mean voxel-wise images. Considering the right CPD, a clear complete duplication was visible, with two well-defined gyri (Supplementary Figure 1b, right hemisphere). However, the mean images of the 48 individuals having a left CPD, obtained after registration in the Fsaverage default surface template exhibited a loose definition of the second gyrus (Supplementary Figure 1b, left hemisphere) upon visual inspection, which was very different from what was observed in the mean images of the same group deriving from VBM (Supplementary Figure 1a, left hemisphere).

The discrepancy observed by visual inspection between the mean images of left CPD obtained with VBM and SBM processing prompted us to develop a specific surface-based template to optimize the inter-subject surface-based normalization of HG by taking into account the distribution of the duplications in our sample. We therefore computed a specific surface-based template (the 40-average SBM template) including images from individuals with identical HG duplication patterns in the left and right hemispheres to avoid a left alignment weighted towards the single HG scenario and a right alignment weighted towards the duplication scenario. Forty individuals were included (26 men, 18 right-handers), corresponding to 20 bilateral single HG and 20 bilateral duplications (10 CSD and 10 CPD). The procedure for computing this specific surface-based template is illustrated in supplementary Figure 2.

*Statistical comparison of groups with varying HG duplication patterns with FSaverage default surface-based inter-subject registration*

The statistical analyses presented in this study were performed on images that have been registered to the 40-average SBM template. The same group comparisons were also performed on the images that had been normalized on the FSaverage default SBM template to evaluate whether the use of different SBM templates modulate the statistical results.

A second analysis of CT variations between left CPD and single left HG has been conducted using a 5 mm surface-based smoothing FWHM Gaussian filter of CT maps instead of 10 mm.

**Results**

*Impact of the “40-average template” on HG duplication pattern anatomy*

The visual inspection of the mean image of left CPD after surface-based registration on the “40-average template” showed a qualitative improvement of the macroscopical anatomy of the left CPD compared to the registration on the Fsaverage default template (Supplementary Figure 1b and 1c). More precisely, the 2^nd^ posterior HG was better defined and was two times larger than that obtained with the Fsaverage default template, and was thus closer to the mean image of the CPD group obtained with the VBM procedure (Supplementary Figure 1a).

Vertex-wise analyses of anatomical differences between groups established according to their HG gyrification pattern were also performed with individual maps registered in the default template namely Fsaverage (Supplementary Figure 3). Except the disappearance of a small cluster of CSA on the left in the comparison between CPD and single HG that was present in the analysis performed after registration on the 40-average SBM template (see Figure 3B) and is absent in the present results obtained after registration on Fsaverage, results were similar in terms of significance, location and cluster size. It suggests that the SBM method is very robust and does not depend on the template used.

We reproduced the analysis of CT variations between left CPD and left single HG using a surface-smoothing kernel size two times smaller (i.e. 5 mm) in 1- the default template, and 2- the optimized template. This contrast was chosen because of the previous demonstration that the computation of a mean image from subjects with a left CPD after registration of those individuals on the default template was not optimal. Clusters of CT detected by these two analyses performed separately with each template (*p* < 0.05 FDR) were very close to those detected by analyses performed with the surface-smoothing size of 10 mm in terms of localization and significance, although the extent was slightly smaller. There was no impact of the template on the results provided by analyses performed using a smoothing filter of 5 mm.

**Discussion**

The development of a specific surface-based template aimed at enhancing the inter-subject registration provided a more accurate localization of the structural variations associated with the different HG gyrification patterns. The averaging of the images from individuals with a left CPD after their registration to the default surface-based template (Fsaverage) did not show two distinct gyri, while it was highlighted in VBM registered images, but only showed a small lateral stem of a second gyrus (see Supplementary Figure 1b). Considering that the Fsaverage default surface-based template is constructed from the general population, it is very likely that it includes a high proportion of single HG on the left (66% of the 430 participants from our sample, Marie et al., 2015). The use of this default surface-based template could bias individual HG anatomy towards a single HG due to the alignment procedure in the left hemisphere, and, consequently, be less representative of the duplication patterns. On the contrary, in the right hemisphere, there is a balanced proportion of duplications and single gyrus in the general population (56% have a single gyrus, Marie et al., 2015). The mean images from the right HG are thus likely less weighted towards a single HG and more representative of the different configurations (single and duplication), preventing bias in the inter-subject registration procedure in the right hemisphere. Actually, in the right hemisphere, the mean images of the CPD group registered with the volumetric VBM template, and the mean images averaged after registration on the default surface-based Fsaverage template or our specific 40-average surface-based template were very similar (see Supplementary Figure 1a, 1b and 1c, right part). In contrast, the visual inspection of the mean images of the left CPD obtained with the 40-average template (Supplementary Figure 1c) showed an improvement in the second gyrus definition as compared to mean image computed on images registered on the Fsaverage template (Supplementary Figure 1b). The mean image of the left CPD obtained with the 40-average template was very comparable to the mean image from the VBM approach. Although we did not statistically quantified the improvement related to the definition of the specific 40-average template compared to the default Fsaverage because of the different surface model representations of the two surface-based templates, we hypothesize that the enhanced proportion of duplications in the left 40-average template improved the inter-subject registration of the CPD sample.

However this qualitative improvement (i.e. mean images of normalized individual surfaces on the 40-average template being more respectful of the individual HG anatomy manually identified than mean images computed after normalization on Fsaverage) did not translate into improved sensitivity of the statistical results. The results obtained with individual images registered on the 40-average template (Figure 3) were very close to those obtained with images normalized on the FSaverage default surface-based template (Supplementary Figure 3). Indeed, the mean curvature images (computed using the FSaverage or the specific 40-average templates) have a spatial resolution close to that of the native MRI images (close to 1 mm^3^ resolution). At this high spatial resolution the reduction of the residual inter-subject variability after spatial normalization onto a specific surface-based template in regions surrounding the HG leads to an improvement of the mean images (see supplementary Figure 1). On the other hand, the group comparisons are performed at lower spatial resolution because of the smoothing procedure completed before the statistical analysis (10 mm surface-smoothing of the CT and CSA maps). This smoothing process, by decreasing the spatial resolution, could diminish the gain in curvature alignment provided by the specific template. This might likely explain the absence of difference between the analyses performed with the different surface-based templates. Finally, the two fold decrease of the surface-based smoothing filter size (from 10 to 5 mm) did not impact the statistical differences detected between left CPD and left single HG, demonstrating the consistency of the results we obtained with SBM.

**Figure legends**

**Supplementary Figure 1. Mean images of groups varying in gyrification pattern after Voxel-Based Morphometry and Surface-Based Morphometry processing**. a) VBM processing was completed using the BIL&GIN template constituted of 80 healthy volunteers of the sample. The supratemporal plane view was obtained using the knife-cut method corresponding to the generation of an oblique slice passing through HG and parallel to the Sylvian fissure. b) SBM processing was completed using the “Fsaverage” template. c) SBM processing was completed using our specific “40-average space”. For the two SBM processing, the rendering of the mean curvature parameters is superimposed on the supratemporal patch described previously, with gyri in red and sulci in blue, (HG: Heschl’s gyrus; CSD: Common Stem Duplication; CPD: Common Posterior Duplication; L: left; R: right; N: number of averaged hemispheres).

**Supplementary Figure 2.** Flowchart of the iterative algorithm used for the computation of our specific “40-average space” used for the surface-based inter-subject alignment.

**Supplementary Figure 3.** **Comparison of groups varying in gyrification pattern obtained using the default FSaverage SBM template.** a. Significant variations in cortical thickness. b. Significant variations in cortical surface area, the lateral view of the right temporal lobe is provided to illustrate the increase in cortical surface area located in the mid-part of the STS in the “CSD – single HG” comparison. Significant variations are superimposed on the mean patch of each corresponding group (48 left CPD, 96 right CPD, for the “CPD - single HG” and “CPD - CSD” contrasts, 98 left CSD and 95 right CSD while for the “CSD - single HG” contrast, the lateral view is displayed on temporal lobe patch of the right CSD group (CSA: cortical surface area; CT: cortical thickness; CPD: common posterior duplication; CSD: common stem duplication; HG: Heschl’s gyrus; *p* < 0.05 FDR corrected for multiple comparison; cluster extension threshold: 20 mm^2^; hot color indicates a positive variation; cold color indicates a negative variation; light grey: gyrus; dark grey: sulci).
