# Supplementary figures and images for: Surface-Based Morphometry of Cortical Thickness and Surface Area Associated with Heschl's Gyri Duplications in 430 Healthy Volunteers
Source: Front Hum Neurosci. 2016 Mar 7;10:69. doi: 10.3389/fnhum.2016.00069 (PMC4779901; doi:10.3389/fnhum.2016.00069)

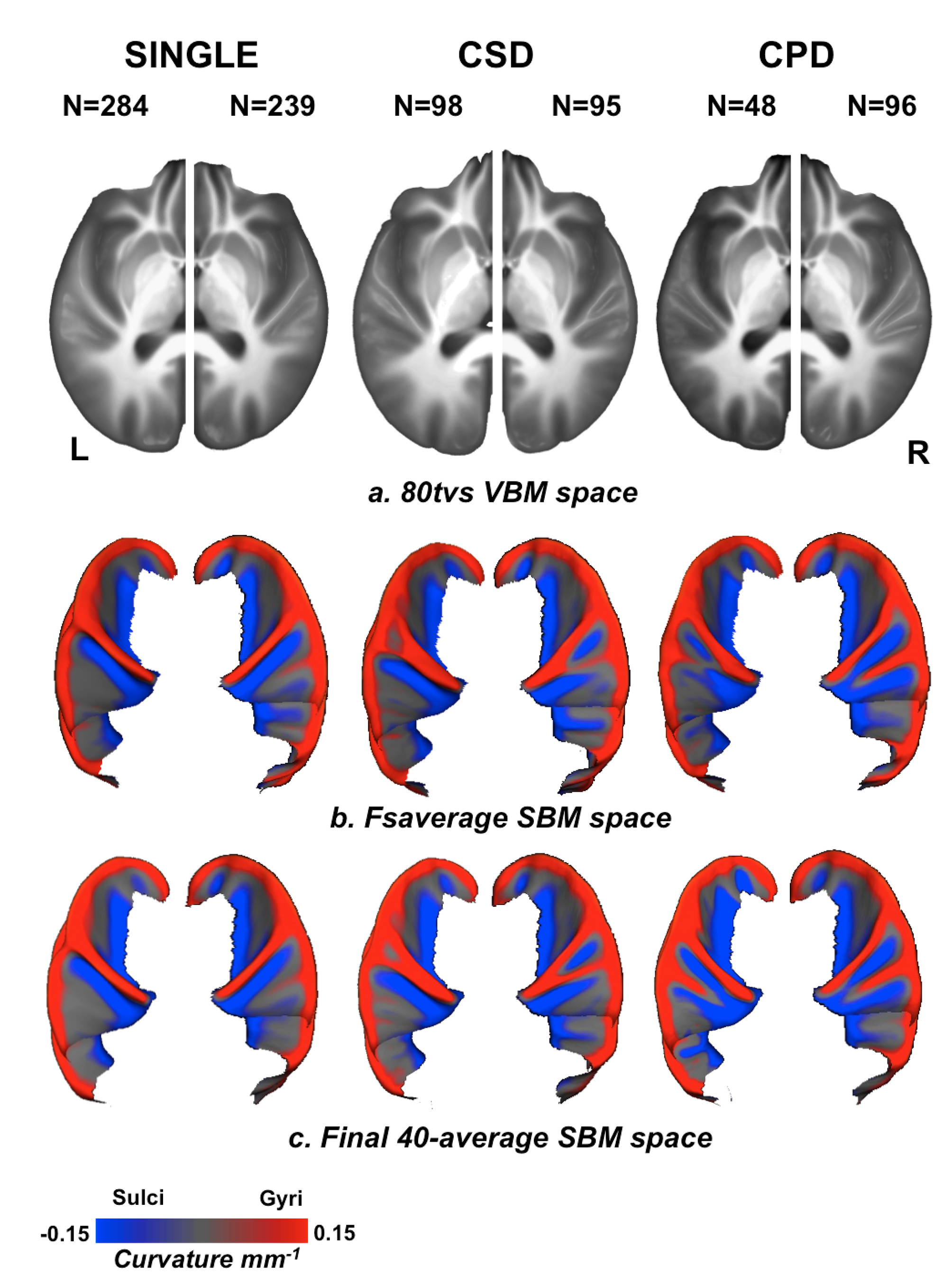

Supplement: Supplementary file 2 [file SupplementaryFigure1.jpeg]

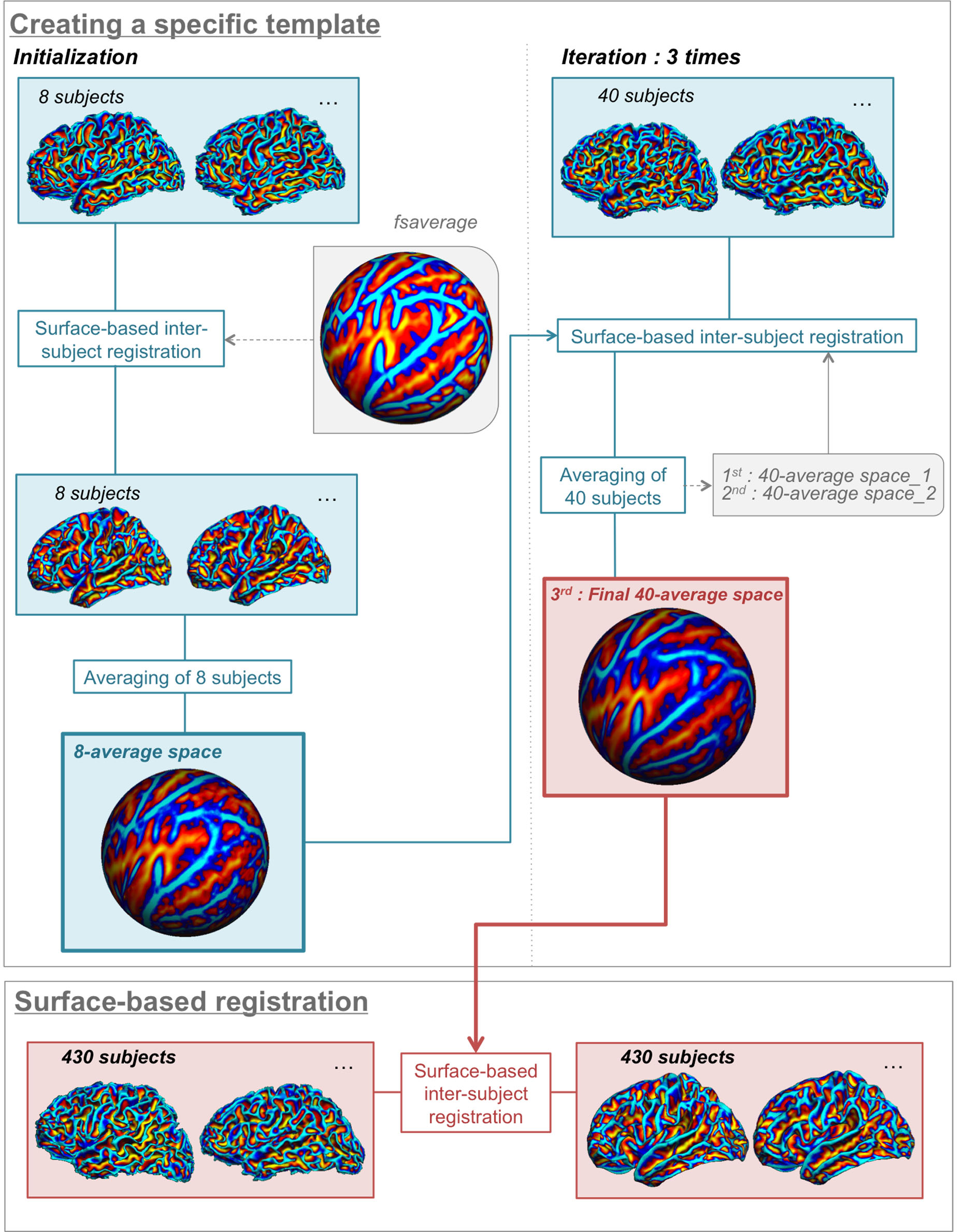

Supplement: Supplementary file 3 [file SupplementaryFigure2.jpeg]

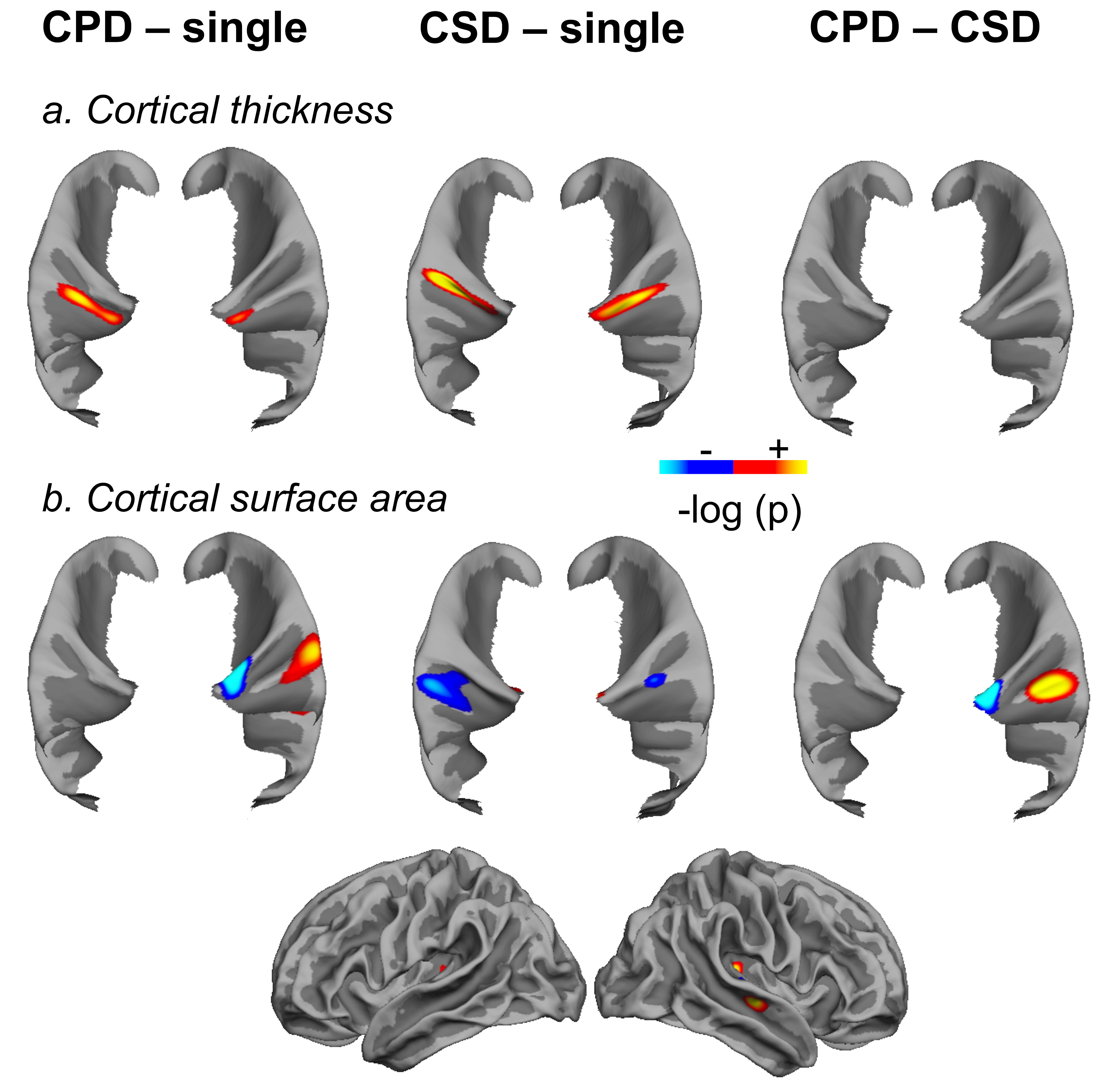

Supplement: Supplementary file 4 [file SupplementaryFigure3.jpeg]
